# A comparative study of shockwave intravascular lithotripsy and conventional percutaneous coronary intervention in the treatment of severe coronary artery calcification lesions

**DOI:** 10.1186/s13019-024-02954-x

**Published:** 2024-07-10

**Authors:** Guang-Xin Zou, Gui-Wu Zhang, Zheng-Dong Wang, Ping Li, Wen-Chao Xie, Jian Chen

**Affiliations:** Department of Cardiology, Yulin First People’s Hospital, The Sixth Affiliated Hospital of Guangxi Medical University, No. 495 Education Middle Road, Yuzhou District, Yulin, 537000 China

**Keywords:** Adverse cardiovascular event, Balloon dilation angioplasty, Coronary artery calcification, Intravascular lithotripsy

## Abstract

**Background:**

The purpose of this study was to evaluate the effectiveness of intravascular lithotripsy (IVL) in the treatment of severe coronary artery calcification (CAC) lesions.

**Methods:**

In this study, we selected patients diagnosed with severe CAC lesions confirmed by coronary angiography (CAG) who were hospitalized in Yulin First People’s Hospital between December 2021 and December 2022 and required percutaneous coronary intervention (PCI). Using a random number table, we divided all patients into the IVL group and the PCI group in the order of interventional therapy. We compared both groups in terms of the surgical success rate, intraoperative manipulation characteristics, procedural complication, and cumulative incidence of major adverse cardiovascular events (MACE).

**Results:**

(1) There were no differences in the surgical success rate, incidence of MACE, and occurrence of procedural complication between the two groups; (2) Compared with the conventional PCI group, patients in the IVL group used fewer predilatation balloons, and the difference was statistically significant (all *P* < 0.05); (3) Compared with the conventional PCI group, patients in the IVL group had lesser surgery time and lesser radiation time, with lesser proportion of patients who were assisted with stent implantation using coronary artery rotational atherectomy, and this difference was statistically significant (*P* < 0.05); (4) The mean stent diameter and length in the IVL group was greater than those in the conventional PCI group but the difference was not statistically significant (*P* > 0.05).

**Conclusion:**

In this study, we found that IVL was a highly safe and effective procedure in the treatment of severe CAC lesions that did not increase the surgery and radiation time, and it could also reduce the use of predilatation balloons, thus improving the management of CAC lesions. Thus, IVL can be a novel choice in treating severe CAC lesions.

## Background

There is a significant prevalence of cardiovascular disease in China due to the aging population, the rise in the incidence of risk factors including hypertension, diabetes, and hyperlipidemia, and the general improvement in the national lifestyle. With 330 million people living with cardiovascular disease and 11.39 million with coronary heart disease, cardiovascular disease is the leading cause of death among urban and rural residents in China [1]. 

Coronary heart disease is one of the most common cardiovascular diseases [1]. Patients with coronary artery stenosis can develop myocardial ischemia symptoms, such as chest tightness and pain, and changes in the heart structure if the condition is not effectively resolved within a reasonable amount of time. Percutaneous coronary intervention (PCI) is the preferred method to address coronary artery stenosis due to its high safety, low trauma, good outcome, and quick recovery. Coronary artery calcification (CAC) increases the risk of procedural complication and the difficulty of the surgery, leading to difficulty in stent implantation or incomplete stent apposition after implantation.

Intravascular lithotripsy (IVL) is gradually being used in disease treatment [2–4]. In this procedure, sound pressure waves are used to compress and crack the calcified lesion without interfering with the tissue; the energy is optimized to treat the vascular calcification and change the vascular compliance while minimizing the injury, so as to maintain the integrity of the original fiber elastic components in the vascular wall. The purpose of this study was to evaluate the efficacy and safety of IVL in managing severe CAC lesions and to offer guidance for the treatment of CAC.

## Materials and methods

### Study respondents

In this study, we selected patients diagnosed with severe CAC lesions using coronary angiography (CAG) who required PCI and were hospitalized in Yulin First People’s Hospital between December 2021 and December 2022. All patients understood this study and signed informed consent, and our study was approved by the Ethics Committee of Yulin First People’s Hospital.

### Diagnosis criteria for severe CAC

Definition of severe CAC lesion: clear, high-density shadows can be seen in the CAG, whether the heat is beating or not [5]. 

### Inclusion and exclusion criteria

#### Inclusion criteria

1. Males and females who were not pregnant, aged 18–80 years;
2. Patients with evidence of silent myocardial ischemia, stable or unstable angina pectoris, or earlier myocardial infarction;
3. Patients with primary lesions and coronary artery lesions in situ as the target lesion;
4. Patients with the reference vessel diameter of the target lesion being 2.5–4.0 mm and the length ≤ 40 mm (visual inspection);
5. Patients with the degree of target lesion stenosis ≥ 70% or ≥ 50% (visual inspection), with evidence of ischemia;
6. The 0.014″ guide wire was able to pass through the lesion;
7. Clear high-density shadows could be seen whether the heart was beating or not;
8. Patients with the target lesion as the only lesion to be treated in this study;
9. Patients with thrombolysis in myocardial infarction (TIMI) grade 3 of the target vessel before the use of the test instrument (predilatation was permitted);
10. Patients suitable for percutaneous transluminal coronary angioplasty (PTCA) and PCI treatment;
11. Patients or their guardians who understood the objectives of the trial, voluntarily participated in the study, and signed informed consent, and could be followed up.

#### Exclusion criteria

1. Patients with dissection in the target vessel in preoperative angiography or after guide wire crossing;
2. Patients with prior stents implanted within 10 mm of the proximal or distal end of the target lesion;
3. Patients with the target lesion located in or involving the anterior descending artery, circumflex artery, and within 5 mm of the right coronary artery opening;
4. Patients with a left main coronary artery lesion or bypass graft lesion;
5. Patients whose angiography showed that the vascular path was tortuous and the test instrument was difficult to reach the target location or recover;
6. Patients with a history of active peptic ulcer or gastrointestinal hemorrhage within six months before enrollment in the study;
7. Patients diagnosed with malignancy or with a life expectancy of less than 12 months;
8. Patients who developed stroke within six months before enrollment in the study, excluding transient ischemic attack (TIA) and lacunar infarction;
9. Patients with platelet counts < 80 × 109/L;
10. Patients with severe liver and kidney function impairment, with transaminase > 3 times the upper limit of the normal value, creatinine > 2.5 mg/dL (221 µmoI/L), or with chronic renal failure requiring long-term dialysis;
11. Patients in grade III or IV of the New York Heart Association (NYHA) classification; [6].
12. Patients known to be allergic to heparin, contrast agents, aspirin, clopidogrel, or anesthetics;
13. Patients who were participating in clinical trials of other drugs or instruments;
14. Patients who the investigator determined to be noncompliant and unlikely to finish the study as planned.

### Instrument

Generic name: Shockwave coronary artery lithotripsy system (manufactured by Sonosemi Medical Co., Ltd.)

Specification: There were seven models and specifications of Shockwave coronary artery balloons. Detailed parameters are shown in Table 1.

**Table 1:** Specifications of Shockwave coronary artery balloon catheters

Model and specification	Nominal diameter of balloon (Unit: mm)	Nominal length of balloon (Unit: mm)
SI-SC001-25012	2.50	12
SI-SC001-27512	2.75	12
SI-SC001-30012	3.00	12
SI-SC001-32512	3.25	12
SI-SC001-35012	3.50	12
SI-SC001-37512	3.75	12
SI-SC001-40012	4.00	12

Effective catheter length: 1,400 mm;

The instrument supported the 0.014″ (0.36 mm) guide wire and rapid exchange balloon catheters.

The model and specification of the in vivo Shockwave therapeutic instrument was SI-SH001-01.

### Experimental design

All enrolled patients had indications for PCI and were diagnosed with severe CAC lesions using CAG. We divided them into the IVL group and the conventional PCI group using a random number table and in order of interventional treatment, and they were given secondary preventive treatment for coronary heart disease. All procedures were based on the current PCI guidelines, expert consensus, and principles of medical ethics in China.

The severe CAC lesion was pretreated in two ways before stent implantation. The IVL group underwent conventional stent implantation after operation (PTCA predilatation was allowed before and after treatment, and rotational atherectomy (RA) was conducted to assist stent implantation, when necessary), while the conventional PCI group underwent conventional stent implantation after PTCA (RA was conducted to assist stent implantation, when necessary).

Patients were followed up for six months post-surgery to evaluate the medium- and long-term treatment efficacy, and we compared the two groups in terms of the surgical success rate, intraoperative procedure characteristics, procedural complication, and cumulative incidence of major adverse cardiovascular events (MACE).

### Outcome indicators

#### Efficacy endpoint: surgical success rate

At the end of the surgery, CAG confirmed residual stent stenosis ≤ 30%, TIMI grade III of forward flow [3], and no occurrence of MACE during hospitalization.

#### Safety evaluation: cumulative incidence of MACE six months post-surgery

Definition of MACE: includes cardiac death, myocardial infarction caused by the target vessel (myocardial infarction with ST segment elevation or non-ST segment elevation), targeted lesion revascularization, cerebrovascular accident, and stent thrombosis.

#### The number of predilatation balloons

The number of predilatation balloons were counted, and we compared and statistically analyzed the use of predilatation balloons between the two groups.

#### Stent implantation

We compared and statistically analyzed the number, size, length, and other aspects of stents implanted after pretreatment of the CAC lesion between the two groups.

#### Usage rate of RA

We measured the use of RA for assisting stent implantation during operation and statistically analyzed the usage rate of RA in the two groups.

#### Operation time and radiation time

The duration of the entire surgery and the duration of radiation during surgery were measured and statistically analyzed.

#### Incidence of procedural complication

We observed the occurrence of procedural complication such as slow flow/no reflow, coronary artery dissection, perforation, cardiac tamponade, severe bradycardia, and hypotension in the two groups.

### Statistical analysis

In this study, we analyzed the data using SPSS 22.0 statistical software. Measurement data were expressed as mean ± standard deviation (${\bar x}$ ± *s*); those in normal distribution and with homogeneous variance were compared between groups using the independent sample t-test, and those not in normal distribution and open data were compared between groups using the non-parametric rank sum test and were expressed as percentile. Enumeration data were expressed as the number of cases and percentage and compared using the chi-square test. *P* < 0.05 indicated statistical differences.

## Results

### Comparison of basic clinical data between the two groups

All 40 patients enrolled in this study completed the six-month postoperative follow-up, and none of the patients were lost to follow-up. In the end, a total of 40 patients participated in the clinical trial, including 20 patients (9 males and 11 females) in the IVL group and 20 patients (13 males and 7 females) in the conventional PCI group. There were no statistical differences in the basic clinical data between the two groups (*P* > 0.05) (Table 2).

**Table 2:** Comparison of basic data between the two groups

Items	IVL group (*n* = 20)	Conventional PCI group (*n* = 20)	*P* value
Gender (n, %)			0.129
Male	9 (45)	13 (65)	
Female	11 (55)	7 (35)	
Age (year, ${\bar x}$ ± *s)*	67.6 ± 7.08	71 ± 8.20	0.168
Height (cm, ${\bar x}$ ± *s*)	160.17 ± 7.76	162.74 ± 8.93	0.252
Weight (kg, ${\bar x}$ ± *s*)	60.63 ± 8.97	58.86 ± 9.55	0.565
Hypertension (n, %)	15 (75)	13 (65)	0.490
Diabetes (n, %)	5 (25)	3 (15)	0.695
Renal insufficiency (n, %)	2 (10)	6 (30)	0.235
Smoking history (n, %)	5 (25)	9 (45)	0.185
Cardiac function (NYHA grades) (n, %)			0.695
Grade I	17 (85)	15 (75)	
Grade II	3 (15)	5 (25)	
Grade III	0 (0)	0 (0)	
Grade IV	0 (0)	0 (0)	
Left ventricular ejection fraction (LVEF, ${\bar x}$ ± *s*, %)	65.55 ± 6.42	61.62 ± 6.63	0.059
Culprit coronary artery (n, %)			1.000
LAD	18 (90)	17 (85)	
LCX	1 (5)	2 (10)	
RCA	1 (5)	1 (5)	
Lesion location (n, %)			0.628
Proximal	17 (85)	12 (60)	
Mesomere	14 (70)	15 (75)	
Distal	1 (5)	2 (10)	
Target vessel diameter (mm, ${\bar x}$ ± *s*)	3.06 ± 0.33	2.94 ± 0.38	0.323
Current targeted vessel length (mm, ${\bar x}$ ± *s*)	25.40 ± 7.82	22.44 ± 7.47	0.257
Degree of targeted vessel stenosis (%)			0.061
70%≤, < 80%	2 (10)	1 (5)	
80%≤, < 90%	13 (65)	7 (35)	
90%≤, < 100%	5 (25)	12 (60)	
Degree of calcification			-
Severe	20 (100)	20 (100)	

Note: LAD is left anterior descending artery, LCX is left circumflex artery, and RCA is right coronary artery

### Comparison of procedural aspects and complication between the two operations

There were no differences in the surgical success rate between the two groups. The IVL group was significantly lower than the conventional PCI group in the number of predilatation balloons (1.70 ± 1.87, 4.05 ± 1.64, respectively; *P =* 0.000), and the difference was statistically significant (*P<*0.05) .There were no statistical differences between the two groups in the number of stents placed (*P* > 0.05); the IVL group was greater than the conventional PCI group in the mean diameter (3.0 ± 0.34, 2.91 ± 0.38, respectively; *P* = 0.158) and the length (26.54 ± 5.63, 23.77 ± 5.46, respectively; *P* = 0.080) of the placed DES, but the difference was not statistically significant (all *P* > 0.05). The IVL group had a lesser proportion of patients assisted with stent implantation with RA than the conventional PCI group, and the difference was statistically significant (*P* = 0.047). The IVL group was lower than the conventional PCI group in the surgery duration (*P* = 0.036) and duration of radiation (*P* = 0.023), and the difference was statistically significant (all *P* < 0.05). There were no significant differences between the two groups in terms of procedural complication (*P* > 0.05) (Table 3).

**Table 3:** Comparison of surgery-related aspects and complications between the two groups

Items	IVL group (*n* = 20)	Conventional PCI group (*n* = 20)	*P* value
Number of predilation balloons (balloon, ${\bar x}$ ± *s*)	1.70 ± 1.87	4.05 ± 1.64	0.000
Number of stents, M (Q1, Q3)	1 (1, 2)	1 (1, 2)	0.748
Mean stent diameter (mm, ${\bar x}$ ± *s*)	3.0 ± 0.34	2.91 ± 0.38	0.158
Mean stent length (mm, ${\bar x}$ ± *s*)	26.54 ± 5.63	23.77 ± 5.46	0.080
Surgery duration (min, ${\bar x}$ ± *s*)	53.51 ± 22.43	60.35 ± 39.61	0.036
Radiation time (min)	23.6 ± 6.50	28.3 ± 9.40	0.023
Number of patients using RA (case, %)	0 (0)	5 (25)	0.047
Degree of postoperative stent stenosis			0.492
≥ 30%	0 (0)	0 (0)	
20%≤, < 30%	1 (5)	4 (20)	
10%≤, < 20%	1 (5)	1 (5)	
10%<	18 (90)	15 (75)	
Postoperative TIMI flow grades, n (%)			-
Grade 0	0 (0)	0 (0)	
Grade I	0 (0)	0 (0)	
Grade II	0 (0)	0 (0)	
Grade III	20 (100)	20 (100)	
Surgical success rate (case)	20 (100)	20 (100)	-
Complication (case)	2 (10)	1 (5)	1.000
Slow flow/no reflow	1 (5)	1 (5)	
Coronary artery dissection	0 (0)	0 (0)	
Coronary artery perforation/cardiac tamponade	0 (0)	0 (0)	
Severe bradycardia	0 (0)	0 (0)	
Hypotension	1 (5)	0 (0)	

### Occurrence of MACE during hospitalization and follow-up in the two groups

Patients in both groups completed the six-month post-surgical telephone or outpatient follow-up, with no MACE occurring during hospitalization or follow-up (Table 4).

**Table 4:** Occurrence of MACE in the two groups during hospitalization and follow-up (case, %)

Items	IVL group (*n* = 20)		Conventional PCI group (*n* = 20)		Total	*P* value
	During hospitalization	During follow-up	During hospitalization	During follow-up		
All-cause death	0 (0)	0 (0)	0 (0)	0 (0)	0 (0)	-
NFMI	0 (0)	0 (0)	0 (0)	0 (0)	0 (0)	-
TVR	0 (0)	0 (0)	0 (0)	0 (0)	0 (0)	-
Stent thrombosis	0 (0)	0 (0)	0 (0)	0 (0)	0 (0)	-
Cerebrovascular accident	0 (0)	0 (0)	0 (0)	0 (0)	0 (0)	-
Total MACE events	0 (0)	0 (0)	0 (0)	0 (0)	0 (0)	-

Note: NFMI is nonfatal myocardial infarction; TVR is targeted vessel revascularization

## Discussion

Severe calcified lesions, especially those that are twisted, angulated, and diffuse [5], not only make PCI more difficult but also significantly increase the incidence of procedural complication and MACE. There were no significant differences between the two groups in terms of procedural complication and MACE. PTCA is a treatment for severe CAC lesions that requires repeated attempts of several conventional balloon pre dilation, multiple pre dilation, and high-pressure pre dilation. After each pre dilation, there may be problems such as restoring to the original stenosis, balloon rupture, and coronary artery dissection due to vascular elastic contraction or even occlusion.

In the pre-treatment of CAC lesions, combining PTCA with RA can reduce the risk of simple PTCA and improve the success rate of surgery [7]. In this study, we found that the number of pre inflation balloons in the IVL group before stent implantation was significantly lower than that in the traditional PCI group (1.70 ± 1.87 and 4.05 ± 1.64, respectively; *P* < 0.05). Contrary to the current situation where traditional PCI requires the use of multiple pre inflation balloons for stent implantation, IVL can reduce the number of pre inflation balloons and reduce the risk associated with multiple balloon expansions.

It can also reduce the use of medical consumables and the risk of multiple balloon predilatations. In this study, we found that the IVL group had a lower proportion of patients who had stent implantation assisted with RA than the conventional PCI group (*P* < 0.05). In the conventional PCI group, in five patients, it was difficult to perform stent implantation with PTCA alone, and the stent was successfully implanted with repeated rotational atherectomy of the target vessel using a 1.5 mm head at a rate of 160,000–200,000 r/min. In one of the five patients, a stent implanted after RA and PTCA failed to pass through the lesion, and one was damaged, but the stent could also be implanted successfully after treatment. This is consistent with the current conclusion that PTCA in combination with RA can reduce the risk of simple PTCA in the pretreatment of CAC lesions and increase the surgical success rate. It also suggests that IVL alone is effective in pretreating severe CAC lesions, and its combination with RA in the treatment of CAC lesions remains to be studied, which may be related to the principle of IVL: it can break or shatter continuous calcified lesions to improve the vascular compliance so as to provide good vessels for stent implantation.

IVL is the only technique that can effectively treat superficial and deep calcification without damaging normal tube walls. In the Disrupt CAD I study by Brinton et al., [8] the endpoint was the successful operation, that is, the residual stenosis after stent implantation was < 50% and no MACE occurred during hospitalization, while the safety endpoint was that no MACE occurred 30 days after follow-up. Both the primary safety and clinical success endpoints were the same as those in the Orbit II trial I [9], which demonstrated the feasibility of IVL in the human body for the first time. IVL was then constantly researched.

A total of 120 European patients were recruited for the prospective, single-arm, multicenter clinical Disrupt CAD II study [10] proposed in the Transcatheter Cardiovascular Therapeutics (TCT) meeting in 2019, and the results showed that 94.2% of them did not develop MACE, perforation, slow flow/no reflow, or artery dissection, while the incidence of MACE was 7.6% during the 30-day follow-up and 79% of the calcified lesions were confirmed to be ruptured in 47 patients completing optical coherence tomography (OCT). These researchers concluded that IVL was safe, with no reports of severe dissection (D-F), perforation, sudden occlusion, or slow flow/no reflow. In the prospective, single-arm, multicenter Disrupt CAD III study, [11, 12] on all target vessels that were severely calcified, the primary safety endpoint was no occurrence of MACE, and the primary efficacy endpoint was the surgical success rate, that is, the residual stenosis after stent implantation was less than 50% by quantitative coronary angiography (QCA) analysis, and no in-hospital MACE occurred. Both endpoints were compared with the preassigned target performance goal. A total of 384 patients were recruited from 47 centers in the United States, the United Kingdom, France, and Germany, among whom 92.2% and 92.4% achieved the primary safety and effectiveness endpoints, respectively.

Studies have shown that in severe calcified lesions, IVL can help push stents and optimize stent dilation in a safe and effective manner. The use of IVL before stent implantation results in good clinical tolerance and a low incidence of perioperative clinical and CAG complications. The prospective, multicenter clinical Disrupt CAD IV study [13] included 64 patients from eight clinical research centers in Japan. The success rates of instruments and angiography (< 50% or 30%) were 98.4%. The mean stenosis was reduced from 65.8% before treatment to 9.9% after stent implantation, with no vascular perforation, persistent slow reflow, reflow, or acute occlusion and stent thrombosis. Aziz et al. [14] studied 190 patients in whom all lesions were treated by IVL; the success rate was 99% and the incidence of procedural complication was 3%, while MACE occurred in only 2.6% of the cases.

In this study, (1) DESs were successfully implanted in both groups after pretreatment of the CAC lesion, and CAG confirmed that the residual stent stenosis was ≤ 30%, TIMI flow in forward flow was in grade III, and no MACE occurred during hospitalization. There was no difference in the surgical success rate between the two groups, which is consistent with the fact that IVL has a high surgical success rate in the pretreatment of CAC lesions in countries outside China. However, this study was done on a small sample and with a short follow-up period, and hence, further experimental results are needed to evaluate the surgical success rate of IVL. (2) There were no statistical differences in the procedural complication between the two groups (*P* > 0.05). One patient in both groups developed slow flow, and the flow was restored after treatment with nitroglycerin, adenosine, and other drugs, with TIMI grade 3 of distal flow; one patient in the IVL group developed hypotension, and the blood pressure returned to normal after treatment with epinephrine, noradrenaline, and other drugs, but this does not indicate that hypotension is common during IVL, and this event may have been related to the operator’s technical levels or other factors. No severe procedural complication, such as coronary artery dissection, perforation, cardiac tamponade, or severe bradycardia, occurred during the procedure in the two groups, suggesting that IVL used to treat CAC lesions did not increase procedural complication and was safe and feasible, which was consistent with the results of the Disrupt series studies. (3) None of the patients in the two groups developed MACE during the six-month postoperative follow-up, and this finding is consistent with the results of the Disrupt series studies that IVL was associated with a low incidence of MACE in the treatment of CAC lesions, suggesting that IVL was highly safe; further follow-up is needed for evaluating its long-term safety.

According to a horizontal meta-analysis of Disrupt CAD trials [15], IVL was safe and effective in the treatment of severe CAC with a high surgical success rate. Tian et al. [16] studied the use of Shockwave balloons to treat severe CAC lesions for the first time in China and reported that multiple cracks on the calcified lesions caused by energy waves were clearly visible in the OCT images, which confirmed the safety and effectiveness of IVL in the pretreatment of CAC lesions in China. Furthermore, based on the perioperative clinical effect of IVL in the treatment of severe CAC lesions, He et al. [17] found that no severe procedural complication occurred immediately after IVL or after PCI in the 27 patients treated using IVL in five centers in China in 2022, with both the surgical success rate and the clinical success rate reaching 100.0% (33/33) and no in-hospital MACE. This further confirmed that IVL had good safety and effectiveness, a high success rate, and a low incidence of procedural complication and MACE in the pretreatment of CAC lesions. Patients in China also had good clinical tolerance to IVL.

In this study, (1) the IVL group had lower surgery time (53.51 ± 22.43, 60.35 ± 39.61, *P* < 0.05) and radiation time (23.6 ± 6.50, 28.3 ± 9.40, *P* < 0.05) when compared to the conventional PCI group, and the difference was statistically significant. This may be because, when used to treat CAC lesions, the energy from IVL cracks the calcification or transforms it into soft tissue. Furthermore, IVL is easy to use; in contrast, conventional PCI requires multiple times of predilatation and even multiple times of RA for CAC, which is time-consuming. (2) The IVL group had a greater diameter (3.0 ± 0.34, 2.91 ± 0.38, *P* > 0.05) and length (26.54 ± 5.63, 23.77 ± 5.46, *P* > 0.05) of the implanted stent than the conventional PCI group, but the difference was not statistically significant. This may be related to the small sample size of this study and our research being restricted to a single center. Studies with a larger sample size and from multiple centers are needed to confirm that larger DESs can be implanted in the IVL group after pretreatment of CAC lesions.

In conclusion, IVL was feasible to use for the management of severe CAC lesions and was highly safe and effective without increasing the occurrence of procedural complication and MACE; it also reduced the use of predilatation balloons and the duration of surgery and radiation time.

The following are the limitations of this study: (1) This study is a single center, and all selected patients are from Yulin City. The influence of regional factors on the results of this experiment cannot be ruled out; (2) The sample size of this experiment is relatively small; (3) The follow-up time of this study is short.

## Conclusion

We found IVL to be highly safe and effective in the treatment of severe CAC lesions without increasing the duration of surgery and radiation time, while also reducing the use of predilatation balloons in conventional stent implantation, thus helping to manage CAC lesions more effectively.

## Data Availability

All data generated or analysed during this study are included in this article. Further enquiries can be directed to the corresponding author.

## Abbreviations

IVL: Shockwave Intravascular Lithotripsy
CAC: Coronary artery calcification
MACE: Major adverse cardiac events
CAG: Coronary arteriongraphy
PTCA: Percutaneous transluminal coronary angioplasty
RA: Rotational atherectomy
DES: Drug-eluting stents
